# Chemical Composition and Antimicrobial Activity against the *Listeria monocytogenes* of Essential Oils from Seven *Salvia* Species

**DOI:** 10.3390/foods12234235

**Published:** 2023-11-23

**Authors:** Maria Francesca Bozzini, Ylenia Pieracci, Roberta Ascrizzi, Basma Najar, Marco D’Antraccoli, Luca Ciampi, Lorenzo Peruzzi, Barbara Turchi, Francesca Pedonese, Alice Alleva, Guido Flamini, Filippo Fratini

**Affiliations:** 1 Dipartimento di Farmacia, Università di Pisa, Via Bonanno 6, 56126 Pisa, Italy; m.bozzini@studenti.unipi.it (M.F.B.); ylenia.pieracci@phd.unipi.it (Y.P.); guido.flamini@unipi.it (G.F.); 2 Centro Interdipartimentale ‘NUTRAFOOD’, Università di Pisa, Via del Borghetto 80, 56124 Pisa, Italy; barbara.turchi@unipi.it (B.T.); francesca.pedonese@unipi.it (F.P.); filippo.fratini@unipi.it (F.F.); 3 Pharmacognosy, Bioanalysis & Drug Discovery Unit, Faculty of Pharmacy, Free University of Bruxelles, Bld Triomphe, Campus Plaine, CP 205/9, B-1050 Bruxelles, Belgium; basmanajar@hotmail.fr; 4 Orto e Museo Botanico, Università di Pisa, Via Luca Ghini 13, 56126 Pisa, Italy; marco.dantraccoli@unipi.it (M.D.); luca.ciampi@unipi.it (L.C.); lorenzo.peruzzi@unipi.it (L.P.); 5 Dipartimento di Biologia, Università di Pisa, Via Derna 1, 56126 Pisa, Italy; 6 Dipartimento di Scienze Veterinarie, Università di Pisa, Viale delle Piagge 2, 56124 Pisa, Italy; 7 Dipartimento di Scienze Agrarie, Alimentari e Agro-Ambientali, Università di Pisa, Via del Borghetto 80, 56124 Pisa, Italy; a.alleva@studenti.unipi.it

**Keywords:** foodborne disease, GC-MS, sage, Lamiaceae, *Salvia chamaedryoides*, listeriosis, caryophyllene oxide

## Abstract

In recent years, essential oils (EOs) have received interest due to their antibacterial properties. Accordingly, the present study aimed to investigate the effectiveness of the EOs obtained from seven species of *Salvia* on three strains of *Listeria monocytogenes* (two serotyped wild strains and one ATCC strain), a bacterium able to contaminate food products and cause foodborne disease in humans. The *Salvia* species analysed in the present study were cultivated at the Botanic Garden and Museum of the University of Pisa, and their air-dried aerial parts were subjected to hydrodistillation using a Clevenger apparatus. The obtained EOs were analysed via gas chromatography coupled with mass spectrometry for the evaluation of their chemical composition, and they were tested for their inhibitory and bactericidal activities by means of MIC and MBC. The tested Eos showed promising results, and the best outcomes were reached by *S. chamaedryoides* EO, showing an MIC of 1:256 and an MBC of 1:64. The predominant compounds of this EO were the sesquiterpenes caryophyllene oxide and β-caryophyllene, together with the monoterpenes bornyl acetate and borneol. These results suggest that these EOs may possibly be used in the food industry as preservatives of natural origins.

## 1. Introduction

According to the World Health Organization’s (WHO) report, foodborne diseases caused by the consumption of food contaminated by harmful microorganisms represent a growing public health concern with respect to their significant socioeconomic impact. The contamination of foodstuffs can occur at any stage of the food-processing chain, resulting from different types of environmental contamination or unsafe storage and processing practices [1]. This hazardous situation necessitates the use of preservatives in the food industry to improve the safety and shelf-life of products [2]. However, while synthetic additives were preferred in the past for their stability and costs, currently, technological progress, globalization, and economic growth have induced important changes in consumers’ behaviour, whose attention is increasingly directed towards the use of natural products, which are perceived as safer and healthier [2,3]. Among the bioactive substances, essential oils (EOs) have received increasing attention in the food industry for their antibacterial properties. This is attributable to the different mechanisms of action, such as their ability to penetrate inside a microorganism’s cytoplasms, disrupting the phospholipid bilayer of mitochondria and the inner membrane and increasing the cellular permeability to constituents and ions and corrupting lipid–protein interactions in bacterial cells, thus affecting ATP production [4,5].

To date, different studies have evidenced antiproliferative effectiveness against both Gram-positive and Gram-negative bacterial strains of different EOs mainly obtained from species belonging to Lamiaceae, such as *Origanum* L., *Ocimum* L., *Thymus* L., *Lavandula* L., and *Rosmarinus* L. (which are included in the genus *Salvia* L. [2,3,5,6,7,8,9]). Lamiaceae comprises many morphologically diverse plants, which are widely distributed worldwide and able to produce large amounts of secondary metabolites [9]. The species of this family have been used for many years as culinary herbs, and recently, they have been employed as natural food preservatives [8] thanks to their antibacterial and antifungal activities, which are primarily attributable to the volatile compounds constituting their EO [9].

Within this family, many species of the genus *Salvia* have been investigated for their phytochemical composition. The presence of numerous secondary metabolites, mostly volatile, has been reported [10]. Since this genus comprises various plants commonly used as spices and food flavourings, which are renowned for their biological activity [10], the present study aimed to investigate the effectiveness of the EOs obtained from different *Salvia* species against *Listeria monocytogenes* in order to evaluate their potential use in the food industry as alternative food preservatives. *L. monocytogenes* is a Gram-positive bacterium able to contaminate food products, causing a foodborne disease in humans known as listeriosis [11,12]. This bacterium is ubiquitous in nature since it may be found in soil, water, and animal digestive tracts [13], all of which are possible sources responsible for the contamination of foodstuffs. According to the WHO, the ingestion of food contaminated with enough *L. monocytogenes* constitutes the main route of infection [13].

In detail, seven species of *Salvia* (*S. apiana* Jeps., *S. aurita* L.f., *S. chamaedryoides* Cav., *S. dolomitica* Codd, *S. dominica* L., *S. officinalis* subsp. *lavandulifolia* (Vahl) Gams, and *S. namaensis* Schinz) cultivated in the Botanic Garden and Museum of the University of Pisa were studied. The aerial parts, deriving from plant pruning for containment and embellishment purposes, were air-dried and subjected to hydrodistillation with a Clevenger apparatus. The obtained EOs were subsequently analysed via gas chromatography coupled with mass spectrometry (GC-MS) and then tested in triplicate on three strains of *L. monocytogenes* (two field strains phenotypically and genotypically identified, as well as serotyped, and one ATCC strain) according to the MIC (minimal inhibitory concentration) and MBC (minimal bactericidal concentration).

## 2. Materials and Methods

### 2.1. Plant Material

The *Salvia* species, as objects of the present study, were cultivated in the Botanic Garden and Museum of the University of Pisa and belong to a collection certified by the Italian Botany Society as a “Collection of National Relevance” for both the number and biodiversity of the represented specimens. The analysed species and their accession numbers, which are in accordance with the plant documentation system, of the botanic garden are reported in Table 1.

These plants are cultivated on the ground, irrigated once or twice a week during the summer months, and fertilised with organic fertilisers twice a year. Their aerial parts, deriving from pruning operations performed for the accession containment and embellishment, were air-dried at room temperature and in the dark to avoid photo-oxidative reactions.

### 2.2. Essential Oil (EO) Hydrodistillation

The air-dried aerial parts obtained from each species (50–80 g for each of the three replicates) were subjected to hydrodistillation with a standard Clevenger apparatus, for 2 h. A small aliquot of the obtained EO was diluted to 5% in HPLC-grade *n*-hexane and injected into the GC-MS apparatus for chemical analysis, and the remaining amount was stored in a refrigerator at −20 °C before being used in biological analyses.

### 2.3. Gas Chromatography–Mass Spectrometry Analysis

The EOs were analysed by means of gas chromatography-electron ionisation mass spectrometry (GC-EIMS) using an Agilent 7890B gas chromatograph (Agilent Technologies Inc., Santa Clara, CA, USA) endowed with an Agilent HP-5MS capillary column (30 m × 0.25 mm; coating thickness 0.25 µm) and an Agilent 5977B single quadrupole mass detector (Agilent Technologies Inc., Santa Clara, CA, USA). The analytical conditions were set as follows: oven temperature increasing from 60 to 240 °C at 3 °C/min; injector temperature at 220 °C; transfer line temperature at 240 °C; carrier gas helium at 1 mL/min; voltage set at 70 kV. The injection volume was 1 µL, with a split ratio of 1:25. The acquisition parameters were as follows: full scan; scan range: 30–300 *m*/*z*; scan time: 1.0 s. Peak identification relied on a comparison between the retention times with respect to those of the authentic samples, comparing their linear retention indices relative to the series of *n*-hydrocarbons (C6-C25) and a computer matching against commercial (NIST 14 and ADAMS 2007) and laboratory-developed mass spectra libraries built up from pure substances and the components of commercial EOs of known composition and the MS literature data [14,15,16,17,18,19].

### 2.4. Statistical Analysis

The chemical classes and hydrodistillation yield of the EOs were subjected to analysis of variance (ANOVA) to evaluate the presence of statistically significant differences. The occurrence of statistical differences between average values was checked using Tukey’s *post hoc* test (*p* < 0.05).

### 2.5. Listeria Monocytogenes Characterization

Three *L. monocytogenes* strains were employed in the experiments. Two of them, identified as 55A and 559E, were wild isolates obtained from goat cheese and goat brain, respectively, while the third strain, ATCC 7644, which is of human origin, was purchased from Thermo Fisher Scientific (Milan, Italy). The strains were stored at −20 °C in a 15% glycerol suspension. For molecular analyses, the strains were revitalized in Brain Heart Infusion broth (BHI, ThermoFisher Scientific, Milan, Italy) and incubated at 37 °C for 18 h. Subsequently, DNA extraction was carried out using GenEluteTM Bacterial Genomic DNA kits (Sigma-Aldrich, St. Louis, MO, USA) according to the manufacturer’s instructions. The extracted DNA was employed in a multiplex PCR as described by Doumith et al. (2004) [20]. PCR products (5 μL) were separated by electrophoresis (100 V) on 2% aga-rose gels and visualized by GelRed™ (Biotium, Fremont, CA, USA) staining. PCR product sizes were determined by comparison with a Gel-Ready^TM^ 100 pb DNA ladder (Lucigen, Middleton, WI, USA). This technique allowed us to separate the main *L*. *monocytogenes* serotypes into four groups based on the presence of specific gene combinations in their genomes. Econo TaqR PLUS Master Mix was employed (Lucigen) with a final reaction volume of 25 μL. To precisely determine the serotype, results from genomic characterization were coupled with those from serological analyses: antisera MASTR ASSURE antiserum *Listeria* “O” (Mast Group Ltd., Bootle, UK) O I/II; O IV; O VIII; and O IX were purchased and used as recommended by the manufacturer.

### 2.6. Antibiotic Susceptibility Test of L. monocytogenes Strains

A disk diffusion test was performed to evaluate antibiotic sensitivity. After the revitalization of *L. monocytogenes* strains on Triptone Soy Agar (TSA, ThermoFisher Scientific, Milan, Italy) plates, 2–3 colonies were diluted in 2 mL of saline and then homogenized via vortexing to obtain a suspension with turbidity corresponding to the 0.5 point of the McFarland turbidity scale. Using a sterile swab, the suspension was transferred to Muller Hinton Agar plates (MHA, ThermoFisher Scientific, Milan, Italy) with an addition of 5% laked horse blood. Subsequently, nitrocellulose discs containing the following antibiotics were placed on the plates: Penicillin (P), 10 μg; Vancomycin (VA), 5 μg; Erythromycin (E), 15 μg; Tetracycline (TE), 30 μg; Chloramphenicol (C), 30 μg; Trimethoprim-sulfamethoxazole (SXT), 25 μg; Gentamicin (CN), 10 μg; Streptomycin (S), 10 μg; and Meropenem (MEM) 10 μg (Oxoid, Milan, Italy). Plates were then incubated at 37 °C for 24 h under microaerophilic conditions. Results were interpreted according to EUCAST [21].

### 2.7. Antimicrobial Activity of EOs against L. monocytogenes Strains

Each EO was tested for antimicrobial activity against the following strains of *L. monocytogenes*: 55A, 559E, and ATCC 7644. Bacterial strains, stored at −80 °C in a 15% glycerol suspension, were sowed on Tryptic Soy Agar (TSA) (Oxoid, Milan, Italy) and incubated overnight at 37 °C for bacterial cell revitalization. Subsequently, one colony of each culture was inoculated into Brain Heart Infusion (BHI) broth (Oxoid, Milan, Italy) and incubated at 37 °C for 24 h under agitation to obtain freshly cultured microbial suspensions. The MIC (Minimum Inhibitory Concentration) and MBC (Minimum Bactericidal Concentration) values of Eos for each strain were determined using the two-fold serial microdilution method on 96-well microtiter plates according to the protocol described by Wiegand et al. [22], with some modifications previously reported by Fratini et al. [23]. Both assays were carried out in triplicate, and MIC and MBC results were expressed as *v*/*v* and reported as mode values.

The EOs were stored at 4 °C, and before being tested, they were subjected to microbial analysis for quality control: one drop of each EO was, indeed, spread on blood agar plates (Oxoid, Milan, Italy) and incubated at 37 °C for 24 h in order to verify their sterility.

## 3. Results

### 3.1. Essential Oil Composition

The complete chemical composition of the analysed EOs is reported in Table 2. Overall, 133 chemical compounds were identified, covering 83.0 to 99.4% of the compositions.

The EOs of all *Salvia* species in this work showed a prevalence of the class of terpenes, mainly represented by mono- and sesquiterpenes.

Monoterpenes were the most abundant compounds of the EOs of *S. apiana* and *S. namaensis*, and both are characterized by a predominance of the oxygenated form. In more detail, this class accounted for 76.1% with respect to *S. apiana*, and its major compounds were camphor (46.3%) and 1,8-cineole (28.0%). Similarly, *S. namaensis* showed a significant abundance of oxygenated monoterpenes (44.6%), with camphor and 1,8-cineole as key components. Significant amounts of oxygenated sesquiterpenes (30.3%) were also detected; the major compound was τ-cadinol, accounting for almost 10% of the entire composition. *S. chamaedryoides* displayed an intermediate chemical composition, with comparable amounts of mono- and sesquiterpenes in its EO and a prevalence of oxygenated forms. The chief constituent was caryophyllene oxide (oxygenated sesquiterpene), followed by comparable amounts of β-caryophyllene (9.9% sesquiterpene hydrocarbon), bornyl acetate, and borneol (9.2% and 8.0%, respectively, oxygenated monoterpenes). Conversely, all other analysed species exhibited a greater abundance of sesquiterpenes. *S. dolomitica* and *S. dominica* showed a prevalence of the hydrocarbon form, reaching 48.0% and 52.7%, respectively, even though the former presented good amounts of the oxygenated form (30.1%) as well. The major components of both species were β-caryophyllene and aromadendrene; however, discernible differences in their content were detected. Oxygenated sesquiterpenes, instead, constituted the major chemical class of *S. aurita*, representing 56.4% of the composition, showing caryophyllene oxide (16.3%) and humulene oxide II (9.9%) as the leading compounds. Finally, in the EO of *S. officinalis* subsp. *lavandulifolia*, similar amounts of both forms of sesquiterpenes were found since hydrocarbon derivatives covered 24.5%, and oxygenated ones covered 22.2%. Moreover, interesting amounts of mono- and diterpenes were also identified, both of which reached almost 15% of the entire chemical profile. *Epi*-Manool was the only detected volatile compound belonging to the class of diterpenes, and it was also the most abundant of the entire composition of *S. officinalis* subsp. *lavandulifolia* EO.

Concerning hydrodistillation yields, the highest values were obtained from *S. dominica* (1.04%) and *S. apiana* (0.98%), while all other analysed species exhibited productivity between 0.3 and 0.5% *w*/*w*.

### 3.2. Characterization of Strains and the Antibiotic Susceptibility Test

Multiplex-PCR-based typing conducted by Doumith et al. [20] allowed us to attribute strain 55A to the first group, which included serotypes 1/2a and 3a; the strain559E to the fourth group, including serotypes 4b, 4d, and 4e; and the ATCC 7644 strain to the second group, including serotypes 1/2c and 3c. By coupling these results with those deriving from serotyping, we were able to ascribe field strain 55A to serotype 1/2a and 559E to 4b, while ATCC 7644 was ascribed to 1/2c.

Concerning the antibiotic susceptibility profile, the tested strains were susceptible to all antibiotics included in the tests.

### 3.3. Antimicrobial Activity of the EOs

The results concerning antibacterial activity are shown in Table 3 and express the mode values of each EO employed against the three strains of *L. monocytogenes*. In detail, the most effective EO was the one obtained from *S. chamaedryoides*, which provided an MIC value of 1:256 *v*/*v* (3.13 mg/mL) for all *L. monocytogenes* strains, followed by the EOs of *S. dolomitica*, *S. aurita*, and *S. officinalis* subsp. *lavandulifolia*, exhibiting an MIC value of 1:128 *v*/*v* (6.12, 5.57, and 5.92 mg/mL, respectively). In contrast, the mode values of bactericidal activity were 1:64 *v*/*v*, which is still high but lower than those of inhibitory activity. Moderate but interesting inhibitory activity was found for the EO obtained from *S. dominica* (MIC mode value of 12.31 mg/mL), while milder activity was detected for the EOs of *S. apiana* and *S. namaensis*, showing MIC mode values of 27.26 and 14.17–28.34 mg/mL, respectively.

## 4. Discussion

Foodborne diseases represent an important public health issue that strongly affects socioeconomic conditions [1]. Climate change exacerbates this preexisting concern, impacting the global food system and introducing new challenges regarding food safety [24]. Indeed, food safety is connected, both directly and indirectly, to the achievement of many sustainable development goals (SDGs) reported in the 2030 Agenda for Sustainable Development, particularly those related to ending hunger and poverty and promoting good health and well-being [25]. Changes in consumer behaviour, as well as greater awareness of the origins of food, the processing chain, and the impact of food products on human health, have introduced new challenges in terms of safety. Within this evolving context, EOs have gained considerable interest in the food industry with respect to their antibacterial properties, and they could be used as alternatives to synthetic additives in order to obtain safer and less perishable food.

In this study, the antibacterial activity of the EO obtained from seven *Salvia* species against three strains of *L. monocytogenes* was assessed.

Interestingly, the chemical composition of the analysed EOs was not always in line with the data reported in the literature, and these differences could be attributed to various factors, including diverse climatic and environmental conditions, as well as the plant phenological stage and the harvesting time of specimens [26]. According to Krol et al. [26], monoterpenes constitute the major class of compounds in *S. apiana* volatile oil, which, in this work, showed a prevalence of oxygenated derivatives, especially camphor and 1,8-cineole. These compounds were also reported in the previously mentioned work, although the EO primarily featured 1,8-cineole, and camphor was only detected in small amounts, similarly to the findings of Borek et al. [27]. *S. apiana*, also called white sage, is a perennial plant that has been used for a long time by the Native North American Chumash people as a medicinal and ritual plant [28], and its biological activities are due to the volatile secondary metabolites that it contains [26].

*S. namaensis* is a perennial plant used in traditional medicine in the Free State province of South Africa for the treatment of flu symptoms [29]. The EO obtained from the aerial parts of this species in this study showed oxygenated monoterpenes as the main chemical class, with camphor and 1,8-cineole as key components, similarly to *S. apiana*. Additionally, good amounts of camphene and α-pinene were detected, in agreement with Grierson et al. [29] and Fisher [30]. However, in contrast to data obtained from the literature, interesting amounts of oxygenated sesquiterpenes, mainly represented by τ-cadinol, were also detected in the analysed EO. The antibacterials of both *S. apiana* and *S. namaensis* have been reported in the literature [26,29], and they are probably determined by their camphor and 1,8-cineole contents. The EO of *S. chamaedryoides*, a Mexican perennial species [31] with a subshrub habit [32], has not previously been studied for its chemical composition to the best of our knowledge. Few studies have been reported in the literature on the phytochemical composition of *S. chamaedryoides*, and they have been focused on the non-volatile fraction [31]. In contrast to the previously reported *Salvia* species, the EO of *S. chamaedryoides* investigated here showed a predominance of oxygenated terpenes and a slight predominance of sesquiterpenes rather than monoterpenes, with caryophyllene oxide as the most abundant component. However, relevant amounts of β-caryophyllene, bornyl acetate, and borneol were also found. Oxygenated sesquiterpenes also represented the major chemical class of *S. aurita*, constituting more than half of its entire volatile profile, and caryophyllene oxide and humulene oxide II were detected as the most important compounds. *S. aurita* is a South African species widely used in traditional medicine and studied by Kamatou et al. [33] for its antimicrobial, antioxidant, and anti-inflammatory activities. As far as we know, the chemical composition of the EO of *S. aurita* has not previously been investigated. However, Ascrizzi et al. [34] reported the spontaneous volatile emission of fresh leaves and evidenced a predominance of sesquiterpene hydrocarbons, with particular reference to β-caryophyllene. Kamatou et al. [33] highlighted the antimicrobial, antioxidant, and anti-inflammatory properties of *S. dolomitica*, another species native to South Africa [33]. The chemical composition of the EO obtained from this species, showing interesting amounts of sesquiterpenes that are both hydrocarbons and oxygenated, was not congruent with the results reported by Ebani et al. [35] or Kamatou et al. [33], who reported monoterpene hydrocarbons as the major chemical class. These were mainly represented by bornyl acetate and camphor in the former and geraniol and linalyl acetate in the latter. Similarly to the previous species, *S. dominica* EO was predominantly constituted by sesquiterpene hydrocarbons, in contrast to Abdallah et al. [36], who reported that oxygenated monoterpenes, mainly represented by linalool and α-terpineol, were the most abundant class of EOs obtained from both fresh and dried plant material. Finally, the *S. officinalis* subsp. *lavandulifolia* EO analysed in this work showed similar amounts of both hydrocarbons and oxygenated derivatives of sesquiterpenes, aside from the interesting amounts of mono- and diterpenes. *epi*-Manool was the only detected volatile compound belonging to the class of diterpenes and also the most abundant of the entire composition of *S. officinalis* subsp. *lavandulifolia* EO, even though good amounts of α-humulene, camphor, and 1,8-cineole were also detected. The chemical profile of the EO studied herein showed relevant differences compared to the requirements of the International Standardization Organization (ISO) regulation, according to which *S. officinalis* subsp. *lavandulifolia* should contain 10–30% of 1,8-cineole and 11–36% of camphor (ISO 3526:2005) [37].

Concerning antimicrobial activities, the three strains of *L. monocytogenes* were found to be equally sensitive to the same EO regardless of their serotype. The EO that showed the greater inhibitory capacity was that obtained from *S. chamaedryoides*, and this was likely due to its high content of caryophyllene oxide (18.2%), an oxygenated sesquiterpene that is known to exhibit significant antibacterial activity [38]. An interesting level of inhibitory effectiveness of their EOs was also observed for *S. aurita* and *S. dolomitica*. Caryophyllene oxide was once again the major compound in these two species, accounting for 16.3 and 7.6%, respectively, which could explain the antibacterial efficacy of these EOs.

Nevertheless, *S. officinalis* subsp. *lavandulifolia* EO, characterized by very low amounts of caryophyllene oxide (0.9%), featured comparable inhibitory activity. In this case, the remarkable effectiveness of its EO could be due to the action of other leading compounds, such as α-humulene (9.7%), camphor (7.3%), and 1,8-cineole (5.2%), and its antibacterial activity has also been documented in the literature [39,40,41]. Despite the relatively low presence of these components in the EO, the strong inhibitory action against *L. monocytogenes* could be attributed to their synergistic effect. Possible synergistic action could also occur with bornyl acetate, an oxygenated monoterpene reported in the literature for its antibacterial activity [42], which was detected in *S. officinalis* subsp. *lavandulifolia* EO in a greater amount (9.2%) than in the other EOs. In most EOs, it was not detected, with the exception of *S. namaensis*. According to Guimarães et al. [43], EOs with a predominant content of oxygenated terpenes demonstrated greater antimicrobial activity against *L. monocytogenes*, which is probably determined by their ability to form hydrogen bonds with the food matrix [44].

Our results suggest the potential applications of analysed EOs in the food industry in the control of *L. monocytogenes* [45,46]; moreover, EOs could be employed to improve the shelf-life of perishable food, contributing to waste reduction, which currently represents an important challenge for sustainable supply chains [47]. The antimicrobial properties of EOs obtained from different species of *Salvia* are well known, and many studies have focused on their uses in different foods for the purpose of inhibiting or decreasing the development of pathogenic or spoilage microorganisms [48]. In general, EOs have been recognized as promising natural preservatives thanks to their preservative efficacy and safety for human health. However, their practical use in the food system faces some limitations mainly due to their intense aroma, which can affect the organoleptic properties of the product [49]. Currently, to overcome these drawbacks, several technological advancements involving different delivery systems, such as nanoencapsulation, active packaging, and polymer-based coatings, have been developed, which, in turn, improve their bio-efficacy and control the release of EOs [49,50,51,52]. This study is a starting point for future investigations that will evaluate the antimicrobial activity of EOs directly on food products, as well as their use in active packaging. Another point that will be assessed concerns the organoleptic properties of these EOs, which will be considered in order to choose those that best suit various types of food preparations.

## 5. Conclusions

*Salvia* EOs have exhibited good inhibitory activity and antimicrobial properties against *L. monocytogenes*, an important Gram-positive bacterium able to contaminate food products and cause human listeriosis. The effectiveness of these EOs varied among the different species, probably because of their different chemical compositions: In general, greater contents of oxygenated sesquiterpenes seemed to be related to greater antimicrobial capacities. The obtained results suggest possible applications of the analysed EOs in the food industry in different delivery systems, such as nanoencapsulation, active packaging, and polymer-based coatings, which can enhance the bio-efficacy of the EOs while mitigating the challenge posed by their strong aroma.

## Figures and Tables

**Table 1:** Analysed *Salvia* species and their accession number according to the plant documentation system of the Botanic Garden and Museum of the University of Pisa (Italy).

Species	Accession Number
*Salvia apiana* Jeps.	2020-0612/0001
*Salvia aurita* L.f.	2020-0686/0001
*Salvia chamaedryoides* Cav.	2020-0640/0001
*Salvia dolomitica* Codd	2020-0681/0001
*Salvia dominica* L.	2020-0671/0001
*Salvia officinalis* subsp. *lavandulifolia* (Vahl) Gams	2020-0675/0001
*Salvia namaensis* Schinz	2020-0685/0001

**Table 2:** Complete chemical composition of the EOs obtained from the analysed *Salvia* species.

Compounds	l.r.i ^1^	Class	Relative Abundance ± Standard Deviation (*n* = 3)						
			*S. apiana*	*S. aurita*	*S. chamaedryoides*	*S. dolomitica*	*S. dominica*	*S. namaensis*	*S. officinalis* subsp. *lavandulifolia*
tricyclene	922	mh	- ^2^	-	-	-	-	0.2 ± 0.00	-
α-pinene	933	mh	2.7 ± 0.01	3.3 ± 0.03	5.6 ± 0.46	1.5 ± 0.04	2.2 ± 0.08	5.4 ± 0.41	1.5 ± 0.05
camphene	948	mh	3.2 ± 0.16	0.3 ± 0.01	3.5 ± 0.30	0.7 ± 0.04	1.0 ± 0.04	9.1 ± 1.63	3.0 ± 0.04
sabinene	973	mh	-	-	3.1 ± 0.20	-	-	-	-
β-pinene	977	mh	1.1 ± 0.02	1.1 ± 0.04	2.2 ± 0.16	0.3 ± 0.01	0.8 ± 0.01	1.0 ± 0.14	0.9 ± 0.01
myrcene	991	mh	0.5 ± 0.06	0.2 ± 0.01	0.3 ± 0.03	0.4 ± 0.02	0.8 ± 0.01	0.3 ± 0.04	0.4 ± 0.02
*p*-mentha-1(7),8-diene	1004	mh	-	0.2 ± 0.02	-	-	-	-	-
α-phellandrene	1006	mh	-	-	-	-	0.2 ± 0.01	-	-
δ-3-carene	1011	mh	1.3 ± 0.05	-	-	2.1 ± 0.06	2.5 ± 0.04	0.1 ± 0.01	-
α-terpinene	1017	mh	-	-	-	-	0.1 ± 0.02	-	-
*p*-cymene	1025	mh	0.3 ± 0.02	0.6 ± 0.03	-	0.5 ± 0.02	-	0.6 ± 0.11	0.4 ± 0.00
sylvestrene	1027	mh	-	-	-	-	0.2 ± 0.02	-	-
limonene	1029	mh	2.1 ± 0.40	1.7 ± 0.23	1.8 ± 0.10	3.0 ± 0.12	3.7 ± 0.16	0.8 ± 0.09	1.2 ± 0.01
β-phellandrene	1029	mh	-	4.1 ± 0.32	-	-	-	-	-
1,8-cineole	1031	om	28.0 ± 3.81	0.3 ± 0.02	5.2 ± 0.03	6.7 ± 0.22	9.3 ± 0.09	15.2 ± 5.54	5.2 ± 0.14
*(Z)*-β-ocimene	1036	mh	0.6 ± 0.12	0.8 ± 0.01	-	0.5 ± 0.01	1.3 ± 0.03	1.5 ± 0.27	-
*(E)*-β-ocimene	1047	mh	-	0.1 ± 0.00	0.2 ± 0.01	-	0.2 ± 0.01	-	-
γ-terpinene	1058	mh	0.2 ± 0.05	-	0.1 ± 0.01	0.1 ± 0.00	0.4 ± 0.03	0.1 ± 0.01	-
*cis*-sabinene hydrate	1066	om	-	-	0.2 ± 0.01	-	-	-	-
terpinolene	1089	mh	0.3 ± 0.04	-	-	-	-	-	-
*trans*-sabinene hydrate	1098	om	-	-	0.1 ± 0.02	-	-	-	-
linalool	1101	om	0.3 ± 0.03	-	-	-	-	-	-
α-thujone	1107	om	-	-	-	-	-	-	0.9 ± 0.05
β-thujone	1117	om	-	-	-	-	-	-	0.2 ± 0.00
*cis*-*p*-menth-2-en-1-ol	1122	om	-	0.1 ± 0.01	-	-	-	-	-
*trans*-*p*-menth-2-en-1-ol	1139	om	-	0.1 ± 0.01	-	-	-	-	-
*trans*-pinocarveol	1139	om	-	-	0.1 ± 0.01	-	-	-	-
camphor	1145	om	46.3 ± 1.75	0.7 ± 0.02	0.9 ± 0.05	-	-	21.8 ± 4.71	7.3 ± 0.01
borneol	1165	om	0.5 ± 0.03	-	8.0 ± 0.06	2.5 ± 0.08	3.7 ± 0.01	3.0 ± 0.45	1.2 ± 0.07
4-terpineol	1177	om	0.5 ± 0.06	0.2 ± 0.00	0.4 ± 0.01	0.3 ± 0.01	0.3 ± 0.00	0.5 ± 0.08	-
cryptone	1186	nt	-	0.1 ± 0.01	-	-	-	-	-
α-terpineol	1191	om	-	-	-	-	0.2 ± 0.01	-	0.4 ± 0.02
myrtenol	1197	om	-	-	0.1 ± 0.01	-	-	-	-
bornyl acetate	1286	om	0.5 ± 0.11	-	9.2 ± 0.30	-	-	4.0 ± 1.16	-
δ-eIemene	1338	sh	-	-	0.1 ± 0.02	-	-	-	-
α-cubebene	1350	sh	-	-	-	0.2 ± 0.01	0.3 ± 0.00	-	-
eugenol	1357	pp	-	-	-	-	0.1 ± 0.01	-	-
α-ylangene	1371	sh	-	0.1 ± 0.01	-	-	-	-	-
isoledene	1373	sh	-	-	-	0.3 ± 0.02	0.2 ± 0.01	-	-
α-copaene	1376	sh	-	0.4 ± 0.01	-	2.0 ± 0.04	1.7 ± 0.00	0.3 ± 0.04	0.5 ± 0.01
(*Z*)-jasmone	1397	nt	-	-	-	-	0.2 ± 0.01	-	-
α-gurjunene	1410	sh	-	-	-	0.7 ± 0.01	0.6 ± 0.02	0.1 ± 0.02	0.4 ± 0.02
*cis*-α-bergamotene	1416	sh	-	-	-	-	-	-	0.1 ± 0.00
β-caryophyllene	1419	sh	-	3.9 ± 0.12	9.9 ± 0.68	12.0 ± 0.17	19.9 ± 0.26	0.3 ± 0.08	3.0 ± 0.09
β-copaene	1429	sh	-	0.2 ± 0.00	-	0.3 ± 0.00	0.2 ± 0.02	-	-
γ-maaliene	1430	sh	-	0.1 ± 0.00	-	0.6 ± 0.05	0.5 ± 0.01	-	0.2 ± 0.00
β-gurjunene	1433	sh	-	-	-	0.3 ± 0.02	0.1 ± 0.01	-	-
α-maaliene	1438	sh	-	0.2 ± 0.01	-	0.9 ± 0.03	0.7 ± 0.00	-	0.2 ± 0.01
α-guaiene	1439	sh	-	-	-	-	-	0.3 ± 0.10	-
aromadendrene	1442	sh	-	2.1 ± 0.03	-	7.6 ± 0.00	6.3 ± 0.06	-	1.9 ± 0.02
guaia-6,9-diene	1443	sh	0.3 ± 0.09	-	3.3 ± 0.23	-	-	-	-
*iso*germacrene D	1451	sh	-	-	0.2 ± 0.01	-	-	-	-
selina-5,11-diene	1452	sh	-	0.2 ± 0.01	-	1.0 ± 0.01	0.7 ± 0.01	-	0.1 ± 0.00
α-humulene	1453	sh	-	3.5 ± 0.11	0.4 ± 0.03	1.3 ± 0.02	2.0 ± 0.02	-	9.7 ± 0.55
*allo*aromadendrene	1460	sh	-	-	-	0.8 ± 0.01	0.6 ± 0.02	-	0.1 ± 0.01
α-elemene	1462	sh	-	-	-	0.2 ± 0.00	0.1 ± 0.01	-	-
*cis*-muurola-4(14),5-diene	1463	sh	-	-	-	0.1 ± 0.01	0.1 ± 0.00	-	-
γ-gurjunene	1469	sh	-	-	-	0.2 ± 0.01	0.2 ± 0.02	-	-
*trans*-cadina-1(6),4-diene	1474	sh	-	-	-	0.3 ± 0.01	0.4 ± 0.03	-	-
γ-muurolene	1477	sh	-	1.4 ± 0.01	-	0.7 ± 0.01	0.5 ± 0.02	-	0.2 ± 0.00
germacrene D	1481	sh	-	-	0.4 ± 0.06	-	-	0.1 ± 0.02	-
α-amorphene	1482	sh	-	0.1 ± 0.02	-	-	-	-	-
*ar*-curcumene	1483	sh	-	-	-	-	-	-	1.9 ± 0.03
β-selinene	1486	sh	-	0.4 ± 0.00	-	0.4 ± 0.01	0.3 ± 0.03	0.1 ± 0.04	-
δ-selinene	1491	sh	-	-	-	0.3 ± 0.00	0.3 ± 0.01	-	-
valencene	1493	sh	-	1.0 ± 0.02	-	-	-	0.4 ± 0.11	-
viridiflorene	1495	sh	-	-	-	3.3 ± 0.01	4.1 ± 0.06	-	1.3 ± 0.05
bicyclogermacrene	1496	sh	-	-	0.1 ± 0.01	0.7 ± 0.08	-	-	-
eremophilene	1499	sh	-	-	-	0.7 ± 0.00	0.7 ± 0.03	-	-
α-muurolene	1500	sh	-	0.3 ± 0.01	-	0.8 ± 0.01	0.8 ± 0.02	0.2 ± 0.06	0.2 ± 0.00
β-bisabolene	1509	sh	0.1 ± 0.04	-	-	-	-	-	-
*trans*-γ-cadinene	1514	sh	0.3 ± 0.14	1.1 ± 0.01	-	4.5 ± 0.10	4.0 ± 0.01	0.8 ± 0.30	1.5 ± 0.03
cubebol	1515	os	-	0.2 ± 0.01	-	-	-	-	-
*trans*-calamenene	1524	sh	-	1.6 ± 0.09	-	1.4 ± 0.06	0.2 ± 0.04	0.9 ± 0.19	-
δ-cadinene	1524	sh	1.0 ± 0.44	0.5 ± 0.09	-	5.1 ± 0.05	6.5 ± 0.00	1.7 ± 0.35	3.0 ± 0.04
selina-3,7(11)-diene	1530	sh	-	1.5 ± 0.07	-	-	-	-	-
cubenene	1533	sh	-	-	-	0.4 ± 0.01	0.5 ± 0.02	-	0.1 ± 0.00
α-cadinene	1537	sh	-	-	-	0.3 ± 0.00	0.2 ± 0.01	-	-
α-calacorene	1543	sh	-	0.2 ± 0.01	-	0.3 ± 0.00	-	-	0.1 ± 0.01
elemol	1550	os	-	-	0.2 ± 0.02	-	-	-	-
germacrene B	1556	sh	-	0.2 ± 0.00	0.2 ± 0.03	-	-	-	-
ledol	1560	os	-	-	-	0.4 ± 0.01	0.4 ± 0.01	-	0.2 ± 0.00
β-calacorene	1563	sh	-	0.1 ± 0.00	-	-	-	-	-
(*E*)-nerolidol	1564	os	-	-	-	0.2 ± 0.01	0.5 ± 0.03	5.2 ± 2.21	-
maaliol	1566	os	-	0.2 ± 0.01	-	-	-	-	-
palustrol	1568	os	-	-	-	-	-	-	0.3 ± 0.01
spathulenol	1577	os	-	1.9 ± 0.04	0.9 ± 0.04	1.3 ± 0.04	0.8 ± 0.08	-	3.5 ± 0.04
caryophyllene oxide	1582	os	-	16.3 ± 0.05	18.2 ± 0.76	7.6 ± 0.03	4.1 ± 0.09	0.5 ± 0.18	0.9 ± 0.09
globulol	1583	os	-	0.6 ± 0.11	-	0.6 ± 0.12	1.1 ± 0.07	0.5 ± 0.20	1.3 ± 0.10
furopelargone A	1588	os	-	-	1.7 ± 0.18	-	-	-	-
β-copaen-4α-ol	1590	os	-	-	0.5 ± 0.04	-	-	-	-
viridiflorol	1592	os	-	0.8 ± 0.02	-	0.3 ± 0.00	0.2 ± 0.00	-	0.5 ± 0.01
*cis*-β-elemenone	1593	os	-	0.4 ± 0.02	-	-	-	-	-
guaiol	1596	os	-	-	-	-	-	-	0.2 ± 0.01
rosifoliol	1602	os	-	0.4 ± 0.02	-	1.2 ± 0.03	1.2 ± 0.05	-	0.4 ± 0.01
humulene oxide II	1608	os	-	9.9 ± 0.00	0.3 ± 0.01	0.7 ± 0.01	0.3 ± 0.01	0.1 ± 0.06	2.3 ± 0.05
1,10-di-*epi*-cubenol	1615	os	-	0.4 ± 0.01	-	0.3 ± 0.02	0.3 ± 0.02	-	-
1-*epi*-cubenol	1627	os	-	2.6 ± 0.15	2.0 ± 0.07	1.9 ± 0.00	1.1 ± 0.01	3.3 ± 1.53	-
juneol	1628	os	-	-	-	-	-	-	3.9 ± 0.17
γ-eudesmol	1631	os	-	1.7 ± 0.24	-	1.0 ± 0.02	0.3 ± 0.01	0.8 ± 0.45	-
caryophylla-4(14),8(15)-dien-5-ol (unidentified isomer 1)	1633	os	-	2.4 ± 0.16	-	-	-	-	-
caryophylla-4(14),8(15)-dien-5-ol (unidentified isomer 2)	1633	os	-	3.2 ± 0.06	2.0 ± 0.17	0.8 ± 0.09	-	-	-
hinesol	1636	os	-	-	-	0.3 ± 0.03	-	-	-
τ-cadinol	1641	os	3.1 ± 1.42	1.8 ± 0.06	0.4 ± 0.03	4.1 ± 0.07	4.1 ± 0.09	9.9 ± 3.45	-
1,3a-ethano(1H)inden-4-ol, octahydro-2,2,4,7a-tetramethyl	1648	os	-	-	-	-	-	-	1.9 ± 0.13
β-eudesmol	1649	os	2.0 ± 0.85	0.9 ± 0.08	-	2.0 ± 0.09	0.5 ± 0.03	4.1 ± 1.99	-
α-muurolol	1651	os	-	-	-	0.3 ± 0.04	0.3 ± 0.02	-	-
α-eudesmol	1654	os	0.2 ± 0.08	0.8 ± 0.00	-	3.8 ± 0.03	1.6 ± 0.04	4.3 ± 2.12	-
α-cadinol	1655	os	0.6 ± 0.38	1.2 ± 0.06	-	1.9 ± 0.16	1.7 ± 0.13	0.5 ± 0.22	0.8 ± 0.01
pogostole	1655	os	-	1.1 ± 0.02	-	-	-	-	-
*cis*-calamenen-10-ol	1658	os	-	0.5 ± 0.10	-	-	-	-	-
*trans*-calamenen-10-ol	1667	os	-	0.3 ± 0.02	-	-	-	-	-
bulnesol	1668	os	0.2 ± 0.05	-	-	-	-	0.9 ± 0.49	2.4 ± 0.06
14-hydroxy-9-*epi*-(*E*)-caryophyllene	1670	os	-	6.6 ± 0.30	2.3 ± 0.01	1.4 ± 0.13	0.2 ± 0.00	-	0.8 ± 0.03
cadalene	1674	sh	-	0.4 ± 0.03	-	0.2 ± 0.01	-	0.1 ± 0.07	-
aromadendrene epoxide II	1680	os	-	0.2 ± 0.00	-	-	-	-	-
α-bisabolol	1685	os	1.3 ± 0.64	1.5 ± 0.07	-	-	-	0.2 ± 0.05	-
(*Z,E*)-farnesol	1689	os	-	-	-	-	-	-	3.1 ± 0.00
juniper camphor	1694	os	-	0.4 ± 0.01	-	-	-	-	-
benzyl benzoate	1763	nt	-	5.4 ± 0.01	-	-	-	-	-
hexahydrofarnesylacetone	1845	ac	-	0.3 ± 0.01	-	-	-	-	-
isopimara-9(11),15-diene	1907	dh	-	-	-	-	0.1 ± 0.02	-	-
*epi*-manool	2056	od	-	0.3 ± 0.01	-	-	-	-	13.4 ± 1.16
abietadiene	2078	dh	-	-	0.1 ± 0.01	-	-	-	-
kolavelool	2079	od	-	-	-	-	-	-	0.2 ± 0.04
phytol	2112	od	-	0.3 ± 0.05	-	-	-	-	-
methyl sandaracopimarate	2252	od	-	-	0.5 ± 0.06	-	-	-	-
methyl isopimarate	2289	od	-	-	0.1 ± 0.02	-	-	-	-
abietal	2314	od	-	-	3.5 ± 0.26	-	-	-	-
methyl dehydroabietate	2359	od	-	-	0.6 ± 0.08	-	-	-	-
methyl abietate	2377	od	-	-	6.9 ± 0.62	-	-	-	-
abietol	2389	od	-	-	0.6 ± 0.19	-	-	-	-
methyl neoabietate	2431	od	-	-	0.8 ± 0.11	-	-	-	-
Chemical classes			*S. apiana*	*S. aurita*	*S. chamaedryoides*	*S. dolomitica*	*S. dominica*	*S. namaensis*	*S. officinalis* subsp. *lavandulifolia*
Monoterpene hydrocarbons (mh)			12.3 ± 0.26	12.3 ± 0.21	16.8 ± 1.25	9.2 ± 0.29	13.3 ± 0.35	19.1 ± 2.68	7.4 ± 0.09
Oxygenated monoterpenes (om)			76.1 ± 5.40	1.4 ± 0.02	24.3 ± 0.14	9.5 ± 0.30	13.4 ± 0.09	44.6 ± 11.94	15.3 ± 0.28
Sesquiterpene hydrocarbons (sh)			1.7 ± 0.71	19.4 ± 0.35	14.6 ± 1.05	48.0 ± 0.18	52.7 ± 0.35	5.5 ± 1.38	24.5 ± 0.78
Oxygenated sesquiterpenes (os)			7.3 ± 3.41	56.4 ± 0.41	28.6 ± 1.28	30.1 ± 0.77	18.7 ± 0.52	30.3 ± 12.91	22.2 ± 0.26
Diterpene hydrocarbons (dh)			-	-	0.1 ± 0.01	-	0.1 ± 0.02	-	-
Oxygenated diterpenes (od)			-	0.6 ± 0.06	13.1 ± 1.32	-	-	-	13.6 ± 1.20
Apocarotenoids (ac)			-	0.3 ± 0.01	-	-	-	-	-
Phenylpropanoids (pp)			-	-	-	-	0.1 ± 0.01	-	-
Other non-terpene derivatives (nt)			-	5.5 ± 0.01	-	-	0.2 ± 0.01	-	-
Total identified (%)			97.5 ± 1.02	95.7 ± 0.05	97.4 ± 0.12	96.7 ± 0.00	98.5 ± 0.05	99.4 ± 0.33	83.0 ± 0.53
EO hydrodistillation yield (% *w*/*w*)			0.98 ± 0.26	0.32 ± 0.02	0.35 ± 0.09	0.3 ± 0.18	1.04 ± 0.01	0.32 ± 0.13	0.48 ± 0.01

^1^ Linear retention index experimentally determined on an HP 5-MS capillary column; ^2^ not detected.

**Table 3:** Antibacterial activity of the tested *Salvia* EOs against the 3 strains of *L. monocytogenes*.

Essential Oils	Microrganism	Code	MIC a	MIC b	MIC c	MIC Mode	MIC mg/mL	MBC a	MBC b	MBC c	MBC Mode	MBC mg/mL
*S. apiana*	*L. monocytogenes*	55	1:32	1:32	1:32	**1:32**	27.26	1:8	1:16	1:16	**1:16**	54.52
	*L. monocytogenes*	559	1:32	1:32	1:32	**1:32**	27.26	1:16	1:16	1:16	**1:16**	54.52
	*L. monocytogenes*	ATCC 7644	1:32	1:32	1:32	**1:32**	27.26	1:16	1:16	1:16	**1:16**	54.52
*S. aurita*	*L. monocytogenes*	55	1:128	1:128	1:128	**1:128**	5.57	1:64	1:64	1:64	**1:64**	11.14
	*L. monocytogenes*	559	1:128	1:128	1:128	**1:128**	5.57	1:64	1:32	1:64	**1:64**	11.14
	*L. monocytogenes*	ATCC 7644	1:128	1:128	1:256	**1:128**	5.57	1:64	1:64	1:64	**1:64**	11.14
*S. chamaedryoides*	*L. monocytogenes*	55	1:256	1:256	1:256	**1:256**	3.13	1:64	1:128	1:64	**1:64**	12.50
	*L. monocytogenes*	559	1:256	1:256	1:256	**1:256**	3.13	1:64	1:64	1:64	**1:64**	12.50
	*L. monocytogenes*	ATCC 7644	1:256	1:256	1:256	**1:256**	3.13	1:64	1:64	1:128	**1:64**	12.50
*S. dolomitica*	*L. monocytogenes*	55	1:128	1:128	1:128	**1:128**	6.12	1:64	1:64	1:32	**1:64**	12.23
	*L. monocytogenes*	559	1:128	1:128	1:128	**1:128**	6.12	1:64	1:64	1:32	**1:64**	12.23
	*L. monocytogenes*	ATCC 7644	1:128	1:128	1:128	**1:128**	6.12	1:64	1:64	1:64	**1:64**	12.23
*S. dominica*	*L. monocytogenes*	55	1:64	1:64	1:64	**1:64**	12.31	1:64	1:32	1:32	**1:32**	24.61
	*L. monocytogenes*	559	1:64	1:64	1:64	**1:64**	12.31	1:64	1:32	1:32	**1:32**	24.61
	*L. monocytogenes*	ATCC 7644	1:64	1:64	1:64	**1:64**	12.31	1:32	1:32	1:32	**1:32**	24.61
*S. namaensis*	*L. monocytogenes*	55	1:32	1:32	1:32	**1:32**	28.34	1:16	1:32	1:32	**1:32**	28.34
	*L. monocytogenes*	559	1:32	1:32	1:32	**1:32**	28.34	1:16	1:16	1:32	**1:16**	56.68
	*L. monocytogenes*	ATCC 7644	1:64	1:64	1:64	**1:64**	14.17	1:16	1:32	1:8	**1:16**	56.68
*S. officinalis* subsp. *lavandulifolia*	*L. monocytogenes*	55	1:128	1:128	1:128	**1:128**	5.92	1:32	1:64	1:64	**1:64**	11.84
	*L. monocytogenes*	559	1:128	1:128	1:128	**1:128**	5.92	1:64	1:64	1:64	**1:64**	11.84
	*L. monocytogenes*	ATCC 7644	1:128	1:128	1:128	**1:128**	5.92	1:64	1:64	1:32	**1:64**	11.84

The letters a, b, and c represent the single replicates. Bold values indicate the mode of the single replicates results.

## Data Availability

The data used to support the findings of this study can be made available by the corresponding author upon request.
